# Exit, Punishment and Rewards in Commons Dilemmas: An Experimental Study

**DOI:** 10.1371/journal.pone.0069871

**Published:** 2013-08-01

**Authors:** Giangiacomo Bravo, Flaminio Squazzoni

**Affiliations:** 1 Department of Social Studies, Linnaeus University, Växjö, Sweden; 2 Collegio Carlo Alberto, Moncalieri, Italy; 3 Department of Economics and Management, University of Brescia, Brescia, Italy; The University of New South Wales, Australia

## Abstract

Commons dilemmas are interaction situations where a common good is provided or exploited by a group of individuals so that optimal collective outcomes clash with private interests. Although in these situations, social norms and institutions exist that might help individuals to cooperate, little is known about the interaction effects between positive and negative incentives and exit options by individuals. We performed a modified public good game experiment to examine the effect of exit, rewards and punishment, as well as the interplay between exit and rewards and punishment. We found that punishment had a stronger effect than rewards on cooperation if considered by itself, whereas rewards had a stronger effect when combined with voluntary participation. This can be explained in terms of the ‘framing effect’, i.e., as the combination of exit and rewards might induce people to attach higher expected payoffs to cooperative strategies and expect better behaviour from others.

## Introduction

In all cases of public good provision, such as good quality scientific peer review or a clean public beach in a popular place, many individuals are called to pool their private resources for the benefit of the whole group, including those who do not contribute[1]–[3]. In cases of common-pool resource (CPR), such as natural resource exploitation or artificial infrastructure use, individuals benefit by sharing a good where there are significant consumption externalities. In these cases, the problem is not whether to contribute to the common pool but how to reduce the exploitation level from it [4], [5]. Although any collective outcome can be maximised when everyone cooperates, self-interest motivation can induce individuals to free-ride, either by contributing nothing or by extracting more than their sustainable share, predicting that others will do the same.

The archetypal model of these social dilemmas is the public good game (from now on, PG), where participants are endowed with a fixed sum of money and choose whether to keep it in their own private account ( = defection) or to contribute to the public good ( = cooperation) [6], [7]. The amount kept by participants increases their payoff, but contributions are multiplied by a factor 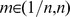 and then divided evenly between everyone, independent of their contribution. Since 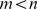, it is not individually beneficial to contribute to the public good, irrespective of what other individuals do. Therefore, the game has a dominant strategy, keeping one’s entire endowment. However, since 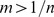, in the case where all participants contribute, everyone would be better off, with a social optimum given by all participants contributing their whole amount.

The experimental results from PG and CPR games consistently rejected the theoretical prediction of universal defection, with cooperation usually starting at intermediate levels. When the game was repeated under anonymity and with no communication, cooperation progressively declined over time, approaching zero after a few rounds [4], [7]. To solve this problem, research looked at a variety of factors which counterbalanced defection, including the marginal cooperation gain (i.e., 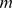), group size and stability, as well as various communication and reputation systems [8], [9].

Furthermore, institutions and organizations provide material and non-material incentives to help people to cooperate [10]–[12]. Various institutional arrangements, such as bonus programmes and ethical codes in organizations, can be seen as targeting interactions which include a coherent set of positive and negative incentives (i.e., rewards and punishments) that can make cooperation more predictable [13], [14]. Similarly, commons management institutions regulate resource exploitation by imposing limits to individual consumption and by punishing overuse [2], [11].

In this respect, certain studies have investigated the effect of both positive and negative institutional incentives [15]–[22]. In this case, any incentive greater than the cost of cooperation, whether positive or negative, should ideally change the dominant defection strategy at an individual level. For instance, imagine a situation where two players simultaneously choose whether to pay a cost 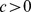 to give a benefit 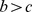 to the opponent. In this case, the structure of the game is similar to a PD with its dominant defection strategy. Nevertheless, both negative and positive incentives, if greater than 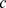, can change a players’ behaviour and lead to full cooperation. Therefore, the mere threat of imposing a fine is sufficient to avoid free-riding, while positive incentives have actually to be paid when cooperation is established, so presenting a direct cost for the organization or institution involved. This has led some authors to argue that negative incentives are more effective than positive ones. This is why both democratic and non-democratic governments largely rely on ‘sticks’ rather than ‘carrots’ to foster rule compliance [23].

Previous research of PG games has shown that individuals are willing to punish defectors even in one shot games or when the possibility of repeated encounters between the same players was ruled out [8], [15]–[18], [22]. Punishment usually takes the form of a fine that subjects can impose on other group members at a cost to themselves. For instance, after receiving information about other players’ behaviour, each participant can decide whether to use part of their endowment to punish other group members or not. In most cases, the rule is that for each monetary unit (MU) used in punishing, the target is fined by three MU [8], [15]–[17]. However, at least in the short run, the cost of fines overcomes any cooperation gains [15], [17], [20]. Although some experiments indicated that a net benefit may be obtained when the interaction is repeated enough [24], punishment decreases participants’ earnings, leaving the question of whether this institutional scheme is actually profitable or robust unsolved. Moreover, recent CPR experiments showed that punishment does not positively affect participant’s earnings unless combined with communication [25].

An alternative to imposing negative incentives on defectors is to give positive ones to cooperators. In this case, participants can use part of their endowment to increase the earnings of other participants, often with the usual three to one ratio. Experiments have shown that participants who cooperate in PG games are inclined to reward other cooperators [19], [20], [22], [26]. Moreover, when possible, participants tend to prefer positive over negative incentives [27]. Although the debate regarding the effectiveness of positive vs. negative incentive is still open, these results indicate that individuals usually prefer not to incur negative sanctions for their behaviour [19], [20], [23], [27].

Furthermore, a few papers have investigated the effect of centralized institutions that might induce participants to cooperate. Attention has so far been concentrated more on understanding the opportunity of implementing these institutional solutions. Kosfeld and colleagues designed a public-good experiment where participants could implement an external cooperation-enforcing ‘organization’ by paying a fixed cost [28]. They found that, even if many groups succeeded in implementing this organization and consequently achieved higher payoffs, this outcome was not robust and depended both on structural factors (e.g., the return rate from the public good and the number of group members) and on the perceived ‘fairness’ of the organization. Similarly, in another CPR experiment, Walker and colleagues found that introducing the possibility of voting for a mandatory ‘allocation rule’ substantially increased outcome efficiency. Surprisingly, they found that requiring unanimity as a condition to select the enforcing institution was more efficient than simply relying on a majority voting rule [21].

Early theoretical works on iterated PD games considered that voluntary participation could led to increased cooperation [29]. Indeed, by introducing an exit option, the predominance of a single strategy was less likely than a rock-paper-scissors succession of cooperators, defectors, and ‘loners’ (agents choosing not to participate in the game) [30]–[33]. In this vein, [34] recently looked at the interplay between incentives provided by an institution and the effect of voluntary participation in public goods games through an applied evolutionary game theory model. Exploring this interplay is key to understanding many empirical situations where there is substantial demand of both positive and negative incentives [35]. Their results indicated that cooperation is less probable when good behaviour is rewarded than where institutional arrangements punish bad behaviour. The combined effect of voluntary participation and positive incentives was weaker, leading to high cooperation levels only when the incentives were considerably higher. By experimentally investigating the interplay and the economic efficiency of positive and negative incentives in public good games, [20] concluded that certain synergies between the two measures could take place, although negative ones are more effective in promoting cooperation and are easier to build in organizations, unfortunately to the detriment of efficiency [23], [26].

This contrasts with the experimental results of [13], where it was found that negative incentives could even reduce cooperation. This was because it induced individuals to frame the game as a self-interest competition situation with defection as the expected dominant strategy, especially in the case of weak and little credible sanctions [36]. By introducing the possibility that players’ identities would be known in repeated public good games, [19] showed that the potential positive incentives could be significantly increased.

Furthermore, while voluntary participation is often considered positive in organizational literature [13], [14], [37], studies on CPR management usually consider any exit option as a factor reducing interdependence within the users’ group and dependence on the resource [38]. This means that an exit can have a negative effect on cooperation in a commons game. Moreover, little is known on the interplay between exit options and incentives [34]. On the one hand, institutional incentives and exit options could be considered as single alternatives, or at best synergistic measures to motivate people and improve individual effort and commitment to cooperation [37]. Indeed, voluntary participation may switch individual attention towards freedom and willingness and so promote self-motivated good behaviour [13], [14], [39]. On the other hand, exits could favour free-riding by allowing individuals to escape negative incentives and by reducing the commitment of individuals towards the group’s interests [40].

The aim of this paper is to shed light on the complex interplay of voluntary participation and incentives, both positive and negative. We experimentally investigated: (i) whether voluntary participation could favour cooperation in commons dilemma situations where incentives are insufficient to establish cooperation, i.e., they do not change the dominant strategy of defection for players, and (ii) what is the effect of the interplay between incentives and the exit option.

## Experimental Design

A total of 144 subjects (58% females) participated in the experiment. They played in sessions of 24 subjects and interacted anonymously through a computer network. Each experimental session took approximate 40 minutes, including instruction reading. The average payoff, including the show-up fee, was 10.52 Euro and all earnings were paid in cash at the end of the experiment.

All participants played 10 periods of an introductory ‘modified public good game’, presented below, plus 10 further treatment periods differing in each session. The goal of the introductory periods was to let the participants familiarize themselves with the game and create a situation where defection dominated, while treatments allowed us to test the effect of the incentive schemes and exit.

The game was played in groups of six, who changed after each period. At the beginning of the game, participants received an endowment of 100 Monetary Units (MU), with an exchange rate of 1 MU = 2 Euro cents. In each period, they were asked to decide whether to bear a cost of 10 MU to provide a benefit of 20 MU to the other group members, evenly divided among them. This meant that the individual payoff in case of full cooperation was 10 MU, while that for full defection was 0 MU. However, a unilateral defector could earn 20 MU while a cooperator in an otherwise defecting group could lose 10 MU. At the end of each period, the resulting payoffs were added/subtracted to/from the endowment and the outcome was communicated to all players.

In designing the game, we followed [34] in assuming that the contribution of each subject provided benefits only to other group members. This meant that contributing in our game was a purely altruistic action, with nothing returned to the cooperative player. This made the decisions unequivocally cooperative and better approximating real-world situations where the direct benefit of cooperation is negligible due to the large number of individuals playing the dilemma.

At the end of the introductory periods, all subjects received a new endowment of 100 MU and played 10 further periods where one of the variables below was manipulated following a 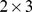 factorial design. The first factor, called *Exit*, was an exit option allowing subjects to decide in each period whether to participate or not in the game, while in the *No Exit* treatments, all subjects played the game as before. Consistent with previous experiments that introduced the same variable, neither exiting or participating in the game had any cost [30], [32]. Subjects who chose to participate played as before, while those who opted out bore no cost but could not derive any benefits from cooperation. Note that, since opting out reduced the number of active group members, each of the remaining players earned a higher share of the 20 MU from cooperative choices. On the other hand, in order to rule out any strategic behaviour from knowing the number of other subjects who participated in the game, each decision to participate and cooperate was taken simultaneously by everyone. To sum up, subjects in the exit treatments faced a three-option choice between (i) providing the benefit, (ii) not providing the benefit, and (iii) not participating in the game. Subjects were also told that, in case of only one subject choosing to participate, the current period game would not be carried out.

The second factor, called *Incentive*, was based on three incentive levels: null, positive, and negative. Under the positive scheme, we assumed that a reward of 5 MU was awarded to cooperators. Under the negative scheme, a fine of 5 MU was dispensed to free-riders. Under the null scheme, all subjects played the game as before. Note that the level of the incentive was intentionally set to ensure that a player’s dominant strategy was still to defect. Here, we assumed all incentives were established by an external controller and that perfect enforcement existed. Table 1 summarizes the six treatments depending on the intersection of all factor levels.

**Table 1:** Overview of the experimental design with treatment labels.

		Incentive		
		Null	Negative	Positive
Exit	No	*No-Null*	*No-Neg*	*No-Pos*
	Yes	*Ex-Null*	*Ex-Neg*	*Ex-Pos*

It is worth noting that while experimental research has mostly examined decentralized punishment [15], [16], [18], in many real-world situations an institution may exist, at least partially separated from individuals or organizations ‘that play the game’, that administers sanctions to players. In cases of private business, public administration and common-pool resources, the puzzle is not who should enforce the rule but whether the enforcement level is effective in providing sufficient incentives to overcome the free-riding temptation of individuals.

### Hypotheses

We formulated six hypotheses about the expected outcome of the different treatments of our experiment (plus the introductory periods).

#### Hypothesis 1

##### In the Introductory Periods, Cooperation is Expected to Start at Intermediate Levels and then Decline

In standard public good games, cooperation usually starts at intermediate levels and then declines. This has been explained in terms of learning or as a reciprocity effect and has been found in many variants of this experiment and similar ones [6], [7], [41]–[43]. There was no reason to expect that our game would be different. Since the cost of cooperation was consistent, i.e., none contribution was returned to the contributor, we expected that the decline could be even more pronounced in our experiment than in standard PG games.

#### Hypothesis 2

##### The No-Null Treatment Should Lead to Cooperation Levels Similar to or Lower than the Introductory Periods. We Expect that its Dynamics Will Follow a Downward Trend

Restarting a public good game usually leads to an increase in cooperation (even if not necessarily up to the initial level) followed by a new decline [44]. In *No-Null*, we expected that cooperation should decline over time, as in the introductory periods. Given that participants had already experienced the game during the introductory periods, we expected that the decline in cooperation could be even more pronounced.

#### Hypothesis 3

##### Cooperation in the Ex-Null Treatment is Expected to be Higher than that Observed in No-Null

Theoretical work [29], [31], [33] and previous experiments have shown that voluntary participation tends to increase cooperation because of the effect of cognitive mechanism such as the ‘projection’ to others of the initial player’s own behavioural intentions [30], [32]. Accordingly, we expected *Ex-Null* to produce a higher proportion of cooperative moves than *No-Null*, where participation was mandatory. This is justified mainly in light of [30], who showed that, when participation is voluntary, intending cooperators are more willing to enter the game than intending defectors. This is because we also expected a higher number of exit choices by intending defectors.

#### Hypothesis 4

##### Positive and Negative Incentives with no Exit (No-Pos and No-Neg treatments) should Increase Cooperation but not Sufficiently to Stop its Downward Trend

Although insufficient in changing the dominant strategy of rational players, we expected that both negative and positive incentives should increase cooperation compared with *No-Null*. This is because individuals tend to react to sanctions even when these are only symbolic [45] or not credible [46]. However, following previous experimental results, we expected that the presence of a significant share of free-riders should progressively reduce cooperation as, for reciprocity reasons, potential cooperators would also be induced to contribute little, with little difference between the *No-Pos* and *No-Neg* cases [16], [20], [43], [47].

#### Hypothesis 5

##### When Exit is Combined with Negative Incentives (Ex-Neg treatment), Cooperation should Increase more than in the No-Null and No-Neg Treatments

Under a negative incentive scheme, free-riders have a rational interest in leaving the game as long as the fees are higher than the expected benefit from the cooperation of other individuals, i.e., 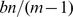 MU, where 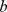 is the benefit given to others group members, 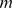 is the number of subjects participating in the game, and 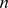 is the number of cooperative subjects. Given the parameters of this game, such an outcome was expected if there were at least two cooperators in the group. The exit of free-riders is expected to allow cooperation to spread at least up to the point where free-riders have a reason to re-enter the game. Moreover, [34] argued that this scheme can lead to full cooperation even with low incentives. We expected to find from intermediate to high levels of cooperation and a significant use of the exit option, mainly by intending defectors.

#### Hypothesis 6

##### When Exit is Combined with Positive Incentives (Ex-Pos Treatment), Cooperation should Increase, but not More than in No-Pos

Under a positive incentive scheme, free-riders have no interest in leaving the game and so the exit option is non-influential. Moreover, Sasaki et al. predicted that this combination should be less effective than the voluntary participation plus negative incentives [34]. Therefore, we expected cooperation levels and game dynamics similar to the *No-Pos* treatment.

## Results

**Figure 1:**
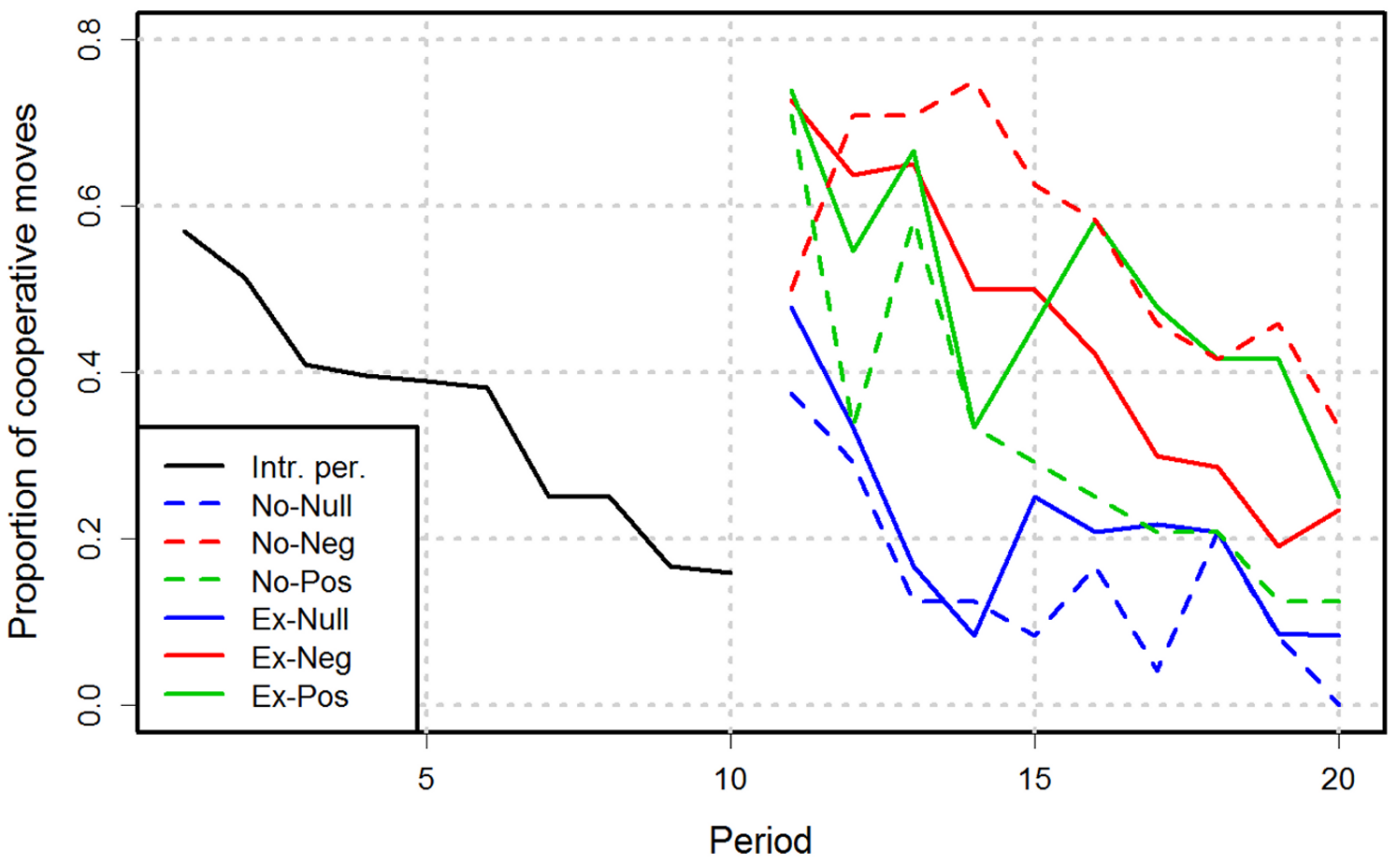
Average cooperation proportion per treatment and period. The introductory period data for all groups were pooled in a single curve.

In line with previous PG game results and consistent with our first hypothesis, cooperation in the introductory periods started at intermediate levels (cooperation proportion in period 1: 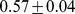) and then declined, leading to a situation where defection was the most common strategy (cooperation proportion in period 10: 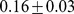). In subsequent periods, cooperation varied depending on the treatment (Fig. 1). Table 2 shows the descriptive statistics for all treatments. All statistical analyses were performed using the *R* platform, version 2.15.1 [48]. The dataset is provided as supporting information.

**Table 2:** Overview of experimental results.

	Female	Participant age		Helping proportion		Exit proportion		Final profit	
Treatment	proportion	mean	sd	mean	sd	mean	sd	mean	sd
No-Null	0.46	23.17	2.90	0.15	0.36	0.00	0.00	115.00	17.11
No-Neg	0.71	23.79	2.62	0.55	0.50	0.00	0.00	133.12	20.32
No-Pos	0.58	23.50	3.58	0.32	0.47	0.00	0.00	147.50	15.99
Ex-Null	0.54	22.08	1.77	0.21	0.41	0.01	0.11	120.83	20.60
Ex-Neg	0.63	22.08	2.32	0.45	0.50	0.15	0.36	115.00	20.56
Ex-Pos	0.58	23.21	2.70	0.49	0.50	0.02	0.13	171.88	18.02
All	0.58	22.97	2.74	0.36	0.48	0.06	0.24	133.89	27.66

**Figure 2:**
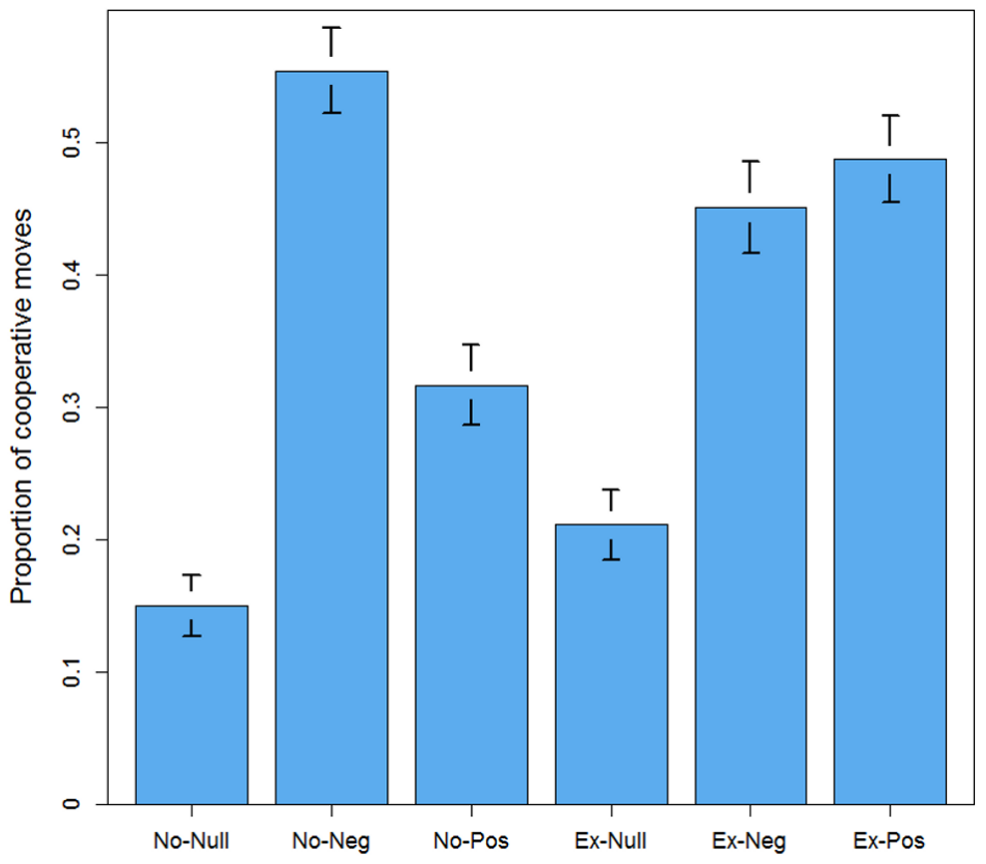
Treatment effects on cooperation. Average cooperation proportion per treatment with standard error bars.

None of the treatments were capable of fully stopping the decline in cooperation typical of PG and social dilemma games. Paired tests on individual averages in periods 1–5 and 6–10 led to the following results: *No-Null*, 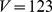, 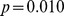; *No-Pos*, 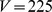, 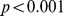; *No-Neg*, 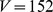, 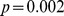; *Ex-Null*, 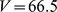, 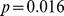; *Ex-Pos*, 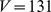, 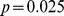; *Ex-Neg*, 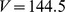, 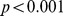 (Wilcoxon signed rank test, 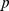 values are one tailed). Nevertheless, our six experimental conditions led to significantly different outcomes (Fig. 2). Consistent with our second hypothesis, defection prevailed in *No-Null*, which was a repetition of the introductory periods and was our control condition. The average proportion of cooperative moves was 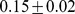 with a declining trend approaching zero cooperation in the final periods.

The introduction of voluntary participation alone did not increase cooperation compared with the previous treatments. Unlike our third hypothesis, *Ex-Null* led to only a minimal increase of cooperation. The average proportion of cooperative moves was 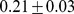, which was not significantly different from *No-Null* (Wilcoxon rank sum test on individual averages: 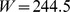, 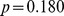 one-tailed). It is also worth noting that participants rarely opted to exit, i.e., only slightly more than 1% of the time.

The outcome changed when institutional incentives were introduced. Although the rewards were theoretically insufficient to alter the players’ dominant strategy, *No-Pos* led to significantly higher cooperation (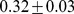) than *No-Null* (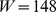, 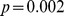 one-tailed), even if defection still dominated, especially in the final periods. On the other hand, *No-Neg* led to a majority of cooperative moves (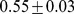). The difference from *No-Null* was highly significant (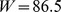, 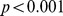 one-tailed) and the treatment led to significantly higher cooperation than *No-Pos* (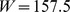, 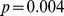 one-tailed). Therefore, in the case of mandatory participation, the influence of negative incentives on cooperation was stronger. This is consistent with our fourth hypothesis, even if the superiority of negative over positive incentives was not expected.

The introduction of voluntary participation combined with the incentive schemes generally led to more cooperation. Consistent with our fifth hypothesis, *Ex-Neg* led to higher cooperation than *No-Null* (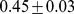, 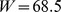, 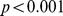 one-tailed). However, cooperation levels were slightly lower than in *No-Neg*, although the difference was statistically significant only at the 10% level (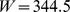, 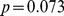 one-tailed). As expected, this was the treatment where most participants chose to exit (15%), with less cooperative participants choosing to exit more frequently, as predicted. The correlation between the individual proportion of cooperative moves in the ten introductory periods of the game and the number of exits in the treatment periods was negative (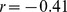). This meant that negative incentives induced intending defectors to seriously consider opting out to avoid fees.

*Ex-Pos* led to a proportion of cooperative moves close to, although somewhat higher than, *Ex-Neg* (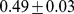). In this case, the subjects rarely chose to exit (i.e., less than 2% of the time). The difference with *No-Null* was highly significant (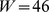, 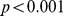). It is worth noting that *Ex-Pos* led to more cooperation than *No-Pos* (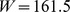, 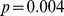 one-tailed). Moreover, unlike the case where participation was mandatory, in this case, the level of cooperation approached the case of negative incentives, i.e., *Ex-Neg*. It is worth noting that, while the fact that *Ex-Pos* led to more cooperation than *No-Null* was consistent with the first part of the sixth hypothesis, the fact that the treatment led to cooperation levels similar to *Ex-Neg* and above *No-Pos* contradicted the second statement of the same hypothesis.

We examined the interplay between the incentives and exits in greater detail by performing an analysis of variance (ANOVA) on the proportion of cooperative moves for each subject in all treatment periods (Table 3). This showed that our factors were overall significant predictors of cooperative behaviour (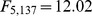, 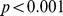). More specifically, the model showed that exit was not significant in itself (so our third hypothesis did not hold) but highlighted a significant interaction effect between exit and positive incentives (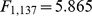, 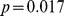) and a weakly significant between exit and negative incentives (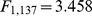, 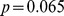). The former effect is consistent with the sixth hypothesis predicting an increase of cooperation when voluntary participation is combined with positive incentives, while the latter supports (at least weakly) the fifth hypothesis on the joint effect of voluntary participation and negative incentives. Moreover, the pure effect of negative incentives was highly significant also considering all interaction effects (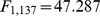, 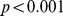), which was a further confirmation of the fourth hypothesis.

**Table 3:** ANOVA table on cooperation (individual averages).

	Df	Sum Sq	Mean Sq	F value	Pr( 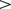 F)
exit	1	0.051	0.051	1.005	0.318
positive	1	0.127	0.127	2.470	0.118
negative	1	2.422	2.422	47.287	0.000
exit×positive	1	0.300	0.300	5.865	0.017
exit×negative	1	0.177	0.177	3.458	0.065
residuals	137	7.018	0.051		

**Figure 3:**
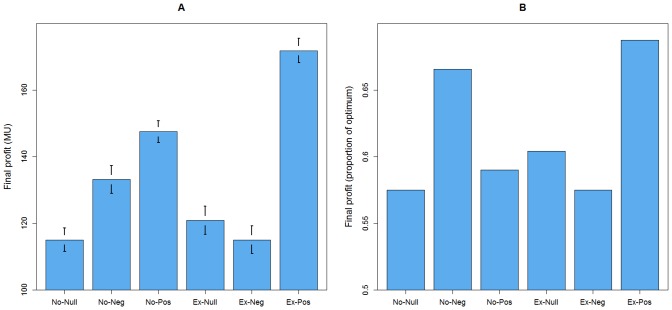
Treatment effects on participants’ profits. (a) Average final profit per treatment with standard error bars. (b) Total profit per treatment as proportion of the optimum.

As regards participants’ earnings, *Ex-Pos* led to the highest absolute final profit, followed by *No-Pos* and *No-Neg* (Fig. 3a). In order to control for the fact that extra money was at stake in *No-Pos* and *Ex-Pos*, we also measured profit as a proportion of the theoretical optimum, i.e., as the amount earned in case of full cooperation plus the sum of all positive incentives (Fig. 3b). In *Ex-Pos*, participants achieved a profit equal to 69% of the optimum, which is the best result of all treatments. This meant that the combination of voluntary participation and positive incentives not only ensured high cooperation but was also economically efficient. In this respect, the second best treatment was *No-Neg* (67%), followed by *Ex-Null* (60%), *No-Pos* (59%), and both *No-Null* and *Ex-Neg* (both 58%).

Additional confirmation of the positive interaction effect between exit and incentives on earnings was in the analysis of variance presented in Table 4. As before, the model significantly predicted differences in earning (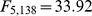, 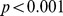). More specifically, this analysis showed that, besides the expected significant pure effect of positive incentives (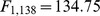, 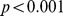), it is worth noting that there were also significant interaction effects between exit and positive incentives (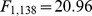, 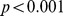) and between exit and negative incentives (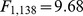, 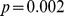), the latter leading to lower average earnings.

**Table 4:** ANOVA table on participants’ final profits.

	Df	Sum Sq	Mean Sq	F value	Pr( 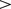 F)
exit	1	584.03	584.03	1.64	0.202
positive	1	47920.92	47920.92	134.75	0.000
negative	1	906.51	906.51	2.55	0.113
exit×positive	1	7452.17	7452.17	20.96	0.000
exit×negative	1	3444.01	3444.01	9.68	0.002
residuals	138	49075.03	355.62		

## Discussion

It is generally acknowledged that individuals are sensitive to the magnitude of incentives and that, when incentives are considerable, cooperation tends to proliferate [49]. However, institutions do not always succeed in providing sufficient incentives to avoid free-riding temptations. This fact motivated us to examine a situation where a sanctioning system existed but was not sufficiently strong to change the dominant strategy of the players. Following recent theoretical investigations, we added voluntary participation in the the game as a second factor potentially capable of increasing cooperation levels [29], [31], [33], notably in interaction with institutional actions [34].

Our experiment confirmed the strength of negative incentives in motivating cooperation, while positive ones led only to small improvements if considered individually. We found that although sanctions were theoretically insufficient to alter the subjects’ rational preferences, *No-Neg* produced a prevalence of cooperation. This contrasted with the idea of a detrimental effect of sanctions on human altruism [50] and more generally, with the idea that monetary incentives can crowd out intrinsic motivations [51], [52].

Indeed, we found that even the imposition of small fines led to a significant increase in cooperation. A possible explanation is that fines might have triggered positive behaviour by highlighting misbehaviour. Although our experimental instructions were abstract and simplified (e.g., by using ‘incentives’ and ‘disincentives’ instead of ‘rewards’ and ‘fines’; see Materials and Methods), it is possible that by penalizing noncooperative action, subjects framed the game as a moral decision and were induced to cooperate more than rationally expected, even if this led to lower earnings.

In contrast with our third hypothesis and with certain previous studies [30], [32], voluntary participation did not increase cooperation if individually considered. This was due to the fact that, participation being a voluntary decision, intending defectors were not motivated to opt out and, therefore, there was no room for a cooperative equilibrium. Even if this happens in many real-world situations, an interesting extension of our study could be to introduce a participation cost or conversely, a fixed reward for non-participation. More generally, our experimental design could be extended to test more complex forms of incentives.

While voluntary participation did not improve the situation by itself, it produced a significant increase of cooperation when coupled with positive incentives. This finding is consistent with [34], who argued that a positive interplay between institutional incentives and voluntary participation could exist. However, we could not support their hypothesis on the superiority of negative over positive incentives. Note that this difference may be due to the fact that, in order to simplify the game structure in a set of understandable instructions, we introduced fixed incentives and assumed that their magnitude did not depend on the number of cooperators and defectors in the population.

It is worth noting that the significant cooperation level in *Ex-Pos* was not due to intending defectors’ choosing not to participate in the game. Indeed, these players had no rational incentive to abstain from playing and actually chose to exit only in a few cases. This could be explained in terms of a ‘frame effect’ [13]: combined with exit, not only did positive incentives induce subjects to expect that only well-intentioned subjects would have participated, but, more importantly, this induced subjects to attach higher expected payoffs to cooperative strategies and predict more cooperation from other subjects.

When considering the aggregate benefit of players, *Ex-Pos* had the highest earnings, both in absolute terms and considering the extra money provided by the institution itself. This result suggests that institutions and organizations could improve their performance by setting up positive incentives while giving individuals the chance of voluntarily choosing whether to participate. Indeed, this could have a frame effect coherent with the positive nature of the incentive and induce individuals to expect more good behaviour from others. This is what happens with voluntary participation of individuals to civic or non-profit associations and organizations, where a good mix of voluntarism and rewards (mostly symbolic) tends to ensure high levels of cooperation that would be unattainable only with rewards or punishment. This could also help to reconsider the conventional approach to public policy, which is presently restricted only to incentives.

To sum up, although weakly significant in itself, voluntary participation led to increased cooperation in commons dilemmas when combined with institutional enforcement. Obviously, in the case of real organizations and institutions, there is no perfect monitoring and some free-riding behaviour may remain unpunished. In this respect, an interesting extension of our work would be to consider monitoring costs and/or asymmetry of information such that subjects could, with a given probability, expect not to be caught. This could lower the relatively good performance of punishment, whereas the negative effect could be less considerable for rewards. However, our results showed that in situations where there is little room for good behaviour, even weak institutionally built-in positive signals for social interaction (i.e., small rewards and voluntary action) can modify the tragedy of the commons.

## Materials and Methods

This section provides additional details on the experiment.

### Ethics Statement

The experiment was held at the University of Brescia on April 23, 2012. Participants were students of the Faculty of Economics recruited using the on-line system ORSEE [53]. All participants were informed and gave their consent when they voluntarily registered to ORSEE. Data collection fully complied with Italian law on personal data protection (D.L. 30/6/2003, n. 196). Under the applicable legal principles on healthy volunteers’ registries, the study did not require ethical committee approval. Participants played in sessions of 24 subjects and interacted anonymously through a computer network running the experimental software z-Tree [54].

### Participant Instructions

The following is the English translation of instructions given to the participants (original in Italian).

### Introductory Periods

#### Screen 1: Overall information on the experiment

- All these instructions contain true information and are the same for all participants.
- Please, read them very carefully. At the end, some questions will be asked by the system to test your understanding of the experiment.
- The experiment you are going to do concerns economic problems.
- During the experiment, you will be asked to take decisions, upon which your final earnings will depend. Earnings will be paid in cash at the end of the experiment.
- Each decision will take place anonymously through your computer screen.
- During the experiment, it is prohibited to talk with anyone. If you do so, you will be excluded from the experiment and you will lose your earnings. Please, turn your mobile phones off.
- For any information and question, put your hands up and wait until an experimenter comes to your position.
- During the experiment, virtual monetary units (MU) are used that have a fixed exchange rate with real Euro.
- For each MU earned in the experiment, you will receive 2 Euro cents.
- For example, if at the end of the experiment your earning is 600 MU, this means that you will receive 12.00 Euro (plus a fixed show-up fee of 5 Euro).

### Screen 2: Interaction Rules

- The experiment consists of a sequence of interaction rounds between groups of 6 players.
- Groups are randomly matched and change each round; therefore, they are made up of different individuals each round.
- There is no way to know whom you are playing with, nor is it possible to communicate with her/him.
- Each participant should make one decision each round.
- The experiment lasts 10 rounds.

### Screen 3: Task Structure

- At the beginning of the experiment, you will receive an endowment of 100 MU. This endowment may subsequently increase or decrease depending on the results of the interaction between your choice and those of the other players.
- In each round, you will have to choose whether to ‘help’ the other members of the group.
- If you choose to help, you will bear a cost of 10 MU, while the other five group members will receive an overall benefit of 20 MU (evenly divided among them).
- If you decide not to help, you will neither bear a cost, nor you will give a benefit to the other group members.
- The choice is free and anonymous. At the end of each round, the aggregate group result will be announced, but not the identity of who decided to help or not.
- All group members must make a choice in each round.
- The benefits will be added to your endowment, while the cost will be deducted from it. Your endowment will be accordingly updated at the end of each round.
- The final endowment represents your earning, which will be converted into Euro with the exchange indicated above (2 cents for each MU).

### No Exit - No Incentives

#### Screen 1: Rules for the continuation of the experiment

- The experiment will now continue for 10 more rounds.
- After these new 10 rounds the experiment will end.
- The MU earned in these new round will be added to your previous earnings.
- The initial endowment for the new rounds is equal to the previous one (100 MU).
- All the game rules remain the same.

### Exit - No Incentives

#### Screen 1: Rules for the continuation of the experiment

- The experiment will now continue for 10 more rounds.
- After these new 10 rounds the experiment will end.
- The MU earned in these new round will be added to your previous earnings.
- The initial endowment for the new rounds is equal to the previous one (100 MU).
- In each of the new rounds, if you do not want to participate in the game, you can do so by pressing the ‘do not participate’ button.
- In the rounds where you decide not to participate, you will neither bear a cost nor give any benefits to the other group members.
- If only one player chooses to participate, the game will not take place and the next round will start.
- All other rules remain the same.

### No Exit - Negative Incentives

#### Screen 1: Rules for the continuation of the experiment

- The experiment will now continue for 10 more rounds.
- After these new 10 rounds the experiment will end.
- The MU earned in these new round will be added to your previous earnings.
- The initial endowment for the new rounds is equal to the previous one (100 MU).
- In the new rounds, who decides not to help will be subjected to a withdrawal of 5 MU to his/her endowment regardless of the other players’ decision to help or not.
- All other rules remain the same.

### Exit - Negative Incentives

#### Screen 1: Rules for the continuation of the experiment

- The experiment will now continue for 10 more rounds.
- After these new 10 rounds the experiment will end.
- The MU earned in these new round will be added to your previous earnings.
- The initial endowment for the new rounds is equal to the previous one (100 MU).
- In the new rounds, who decides not to help will be subjected to a withdrawal of 5 MU from his/her endowment regardless of the other players’ decision to help or not.
- In addition, in each of the new rounds, who does not want to participate in the game can do so by pressing the `do not participate’ button.
- In the rounds where you decide not to participate, you will neither bear a cost nor give any benefits to the other group members.
- If only one player chooses to participate, the game will not take place and the next round will start.
- All other rules remain the same.

### No Exit - Positive Incentives

#### Screen 1: Rules for the continuation of the experiment

- The experiment will now continue for 10 more rounds.
- After these new 10 rounds the experiment will end.
- The MU earned in these new round will be added to your previous earnings.
- The initial endowment for the new rounds is equal to the previous one (100 MU).
- In the new rounds, who decides to help will have a bonus of 5 MU, which will be added to his/her endowment regardless of the other players’ decision to help or not.
- All other rules remain the same.

### Exit - Positive Incentives

#### Screen 1: Rules for the continuation of the experiment

- The experiment will now continue for 10 more rounds.
- After these new 10 rounds the experiment will end.
- The MU earned in these new round will be added to your previous earnings.
- The initial endowment for the new rounds is equal to the previous one (100 MU).
- In the new rounds, who decides to help will have a bonus of 5 MU, which will be added to his/her endowment regardless of the other players’ decision to help or not.
- In addition, in each of the new rounds, who does not want to participate in the game can do so by pressing the ‘do not participate’ button.
- In the rounds where you decide not to participate, you will neither bear a cost nor give any benefits to the other group members.
- If only one player chooses to participate, the game will not take place and the next round will start.
- All other rules remain the same.

### Dataset

This section briefly describes the variables included in the dataset, which is separately provided as supporting information.

**id:** participant's unique id number.

**period:** period number: periods 1–10 correspond to the introductory game, periods 11–20 to the treatment.

**intro:** introductory game: 1 = true, 0 = false.

**ex:** exit allowed: 1 = true, 0 = false.

**pos:** positive incentive: 1 = true, 0 = false.

**neg:** negative incentive: 1 = true, 0 = false.

**help:** participant choice: 1 = helped, 0 = did not help, NA = did not participate in the game.

**exit:** participation (exit) choice; 1 = participated in the game, 0 = did not participate in the game.

## Supporting Information

Dataset S1 **Supporting dataset.**
(XLS)
